# Comparison of vasopressin to epinephrine during pediatric in-hospital cardiac arrest: survival and physiologic responsiveness

**DOI:** 10.1038/s41390-025-04374-6

**Published:** 2025-10-04

**Authors:** Morgann Loaec, Garrett Keim, Kathryn Graham, Martha F. Kienzle, Amanda O’Halloran, Lindsay N. Shepard, Sanjiv Mehta, Samridhi Sawhney, Marion Donoghue, Kellimarie Cooper, Todd J. Kilbaugh, Vinay Nadkarni, Alexis A. Topjian, Robert A. Berg, Robert M. Sutton, Ryan W. Morgan

**Affiliations:** 1 Division of Critical Care Medicine, Department of Anesthesiology and Critical Care Medicine, Children’s Hospital of Philadelphia, Philadelphia, PA USA; 2 Resuscitation Science Center, CHOP Research Institute, Children’s Hospital of Philadelphia, Philadelphia, PA USA

## Abstract

**Aim:**

To compare post-epinephrine vasopressin administration versus epinephrine and time to return of spontaneous circulation (ROSC) during pediatric in-hospital cardiac arrest (IHCA), and explore vasopressin’s physiologic effects.

**Methods:**

This single-center, retrospective cohort study (2017–2023) compared vasopressin patients who received ≥1 dose of preceding epinephrine and matched epinephrine-only patients based on age, illness category, and preceding epinephrine dosing. Time to ROSC was analyzed using Cox regression. Vasopressor response was defined as ≥5 mmHg increase in diastolic blood pressure (DBP).

**Results:**

Forty-one matched pairs were analyzed. Median CPR duration was 36.5 [IQR 23, 48] minutes; median time to dose was 14.5 [10.8, 19] minutes. ROSC occurred in 10/41 (24%) vasopressin and 15/41 (36%) epinephrine patients (*p* = 0.34) with no difference in time to ROSC (aHR 0.73 [95% CI: 0.31–1.7]). Vasopressor response occurred in 4/12 (33%) vasopressin and 1/7 (14%) epinephrine patients (*p* = 0.60). Regression discontinuity analysis demonstrated a change in DBP of +2.3 mmHg after vasopressin (95% CI: −11.4, 16.0) and −5.67 mmHg after epinephrine (–15.13, 3.80).

**Conclusion:**

No significant differences were found in time to ROSC or DBP increase between vasopressin and epinephrine given late during CPR. A subset of vasopressin responders suggests further research on intra-arrest DBP response to vasopressin is needed.

**Impact:**

- The use of intra-arrest vasopressin is an understudied topic in pediatric cardiac arrest.
- This study presents a unique comparison of ongoing epinephrine to vasopressin administration, using dose matching to address the limitations of our current vasopressin use.
- We present a novel analysis of physiologic response to vasopressin using the change in diastolic blood pressure.

## Introduction

In-hospital cardiac arrest (IHCA) affects more than 15,000 pediatric patients in the United States annually.^1^ Prior multicenter investigations have established an association between intra-arrest diastolic blood pressure (DBP) and arrest survival.^2,3^ Current pediatric cardiac arrest guidelines recommend the use of epinephrine to augment coronary perfusion pressure (CoPP) through increased aortic DBP to facilitate return of spontaneous circulation (ROSC).^4,5^ However, the physiologic response to epinephrine during pediatric cardiopulmonary resuscitation (CPR) varies, and a poor response is associated with worse outcomes.^6^ Intra-arrest management strategies for those who do not show a physiologic response to epinephrine are limited.^7^

Though vasopressin is not recommended by pediatric CPR guidelines, laboratory studies report a physiologic response to vasopressin among epinephrine non-responders.^8–10^ At high doses, vasopressin acts directly via V1 receptors to induce peripheral vasoconstriction, which in laboratory studies provides similar increases in DBP and CoPP compared to epinephrine.^11–13^ Clinical data regarding pediatric IHCA that included vasopressin administration are limited.^14–16^ A small pilot trial powered for feasibility showed favorable results with the use of an epinephrine plus vasopressin combination, but the physiologic response to treatment remains unexplored.^16^

Our primary aim was to investigate the association between vasopressin administration compared to matched epinephrine administration and time to ROSC in pediatric patients who received at least one prior dose of epinephrine. We hypothesized that vasopressin dosing would be associated with a shorter time to ROSC through an increase in DBP when compared to matched epinephrine dosing. Our secondary aim was to explore the physiologic response to vasopressin during pediatric IHCA, as determined by the change in DBP following dose administration.

## Methods

### Setting and design

This was a single-center retrospective cohort study of pediatric intensive care unit (PICU) and pediatric cardiac intensive care unit (CICU) IHCAs with prospectively collected event data, including physiologic data, in an institutional cardiac arrest database at a quaternary pediatric care center. This study was approved with a waiver of informed consent from the Children’s Hospital of Philadelphia (CHOP) Institutional Review Board (11-008115).

### Data collection

The CHOP Resuscitation Database is a prospectively enrolling registry of all patients who receive CPR at CHOP or who receive post-cardiac arrest care at CHOP since January 1, 2017. Trained research coordinators prospectively collect demographic and clinical data, including the timing and dose of all medications administered during CPR. Dose times to the nearest minute are collected from paper and electronic code records. Bedside monitor arterial line waveform data are captured for each event through BedMaster (Excel Medical, Jupiter, FL). Arterial waveform data are then reconstructed into an analyzable format using a custom code (MATLAB, The Mathworks, Inc., Natick, MA) and individually analyzed by the investigator team to annotate periods of CPR, non-sustained ROSC, and sections of non-analyzable arterial waveform data. Following this annotation, periods of CPR are analyzed using MATLAB code to determine the compression-by-compression DBP, which is averaged over 15-s epochs of CPR.

### Inclusion and exclusion criteria for the matched cohort

The CHOP Resuscitation Database was queried to identify IHCAs between 2017 and 2023, in either the PICU or CICU. Patients were eligible for inclusion in the vasopressin cohort if they received vasopressin during CPR and received ≥1 dose of epinephrine preceding vasopressin. Patients were excluded if the timing of vasopressin administration was unknown relative to the start time of CPR. For each patient in the vasopressin cohort, patients who did not receive vasopressin during CPR were considered candidates for matching based on the following criteria: (1) were in the same age category (≤1 year, between 1 and 8 years, ≥8 years of age) and hospital illness category (cardiac vs. non-cardiac) as the vasopressin patient and (2) received an additional dose of epinephrine (instead of vasopressin) after the same number of pre-vasopressin epinephrine doses as the vasopressin patient. For example, if a given patient in the vasopressin cohort received three doses of epinephrine followed by a dose of vasopressin, they were matched to a patient in the same age category and with the same illness category who did not receive vasopressin and who received at least four doses of epinephrine. The vasopressin dose, having occurred after three doses of epinephrine, would be matched to the fourth epinephrine dose in the matched patient. Among match candidates, the patient who received the epinephrine dose of interest in the closest minute to the matched vasopressin dose from the start time of CPR was selected. Only one arrest event per patient was eligible for inclusion in the study cohort; however, this did not have to be the index event.

At our institution, vasopressin is available in the ICU locked medication cabinet in the patient care area. Medication dosing booklets available on unit-based code carts recommend a dose of 0.4 units/kg up to 40 units of vasopressin. There are no specific cardiac arrest protocols guiding the use of vasopressin in our institution; the decision to use vasopressin is at the discretion of the individual clinicians/teams.

A subset of patients from this cohort was included in the secondary aim focused on the physiologic response to vasopressin. For inclusion in the physiologic cohort, a patient was required to have at least one evaluable 15-s epoch of invasively measured DBP data in: (1) the 2 min preceding the study dose of vasopressin or epinephrine and (2) the time interval following the study dose (either before the next dose of vasopressor or a maximum of 5 min).

For the analysis of dose responsiveness, the four 15-s epochs of DBP data during the minute of dose administration were excluded from the analysis as time zero (Supplementary Fig. S1). The 2 min of data before the dose were averaged for the pre-dose DBP. The epochs following the study dose until the next dose of vasopressor, or until a maximum of 5 min, were averaged for the post-dose DBP. The pre-dose timeframe was selected to capture an adequate period of time to represent the physiologic state prior to medication administration while minimizing capture of the administration of the prior epinephrine dose. The post-dose measurement window was selected to capture the peak effect of each vasopressor, which occurs within 5 min for both drugs. Prior laboratory-based studies of vasopressin have shown peak effect between 90 s and 4 min after dose administration.^8,12,17^ The difference between the mean post-dose DBP and the mean pre-dose DBP was used to calculate the change in DBP.

### Outcomes

For the primary analysis of vasopressin patients compared to matched epinephrine patients, the primary outcome was time to ROSC from the administration of the study drug dose. For the secondary analysis of physiologic dose responsiveness, the primary outcome was physiologic response to vasopressin or epinephrine, which we defined as an increase in DBP of ≥5 mmHg following the study dose of vasopressor. This threshold was determined a priori based on previous work describing the physiologic response to epinephrine during pediatric IHCA.^6^ Based on this definition, patients were characterized as dose-responders or non-responders.

### Statistical analysis

Categorical variables were summarized as counts and percentages, and continuous variables as medians and first and third quartiles. Fisher’s exact test compared categorical variables and the Wilcoxon rank-sum test compared continuous variables between vasopressin and epinephrine-only patients, and between vasopressin responders and non-responders.

A multivariable Cox proportional hazard regression assessed the association between vasopressin and matched epinephrine administration and time to ROSC. Potential confounders included the primary cause of arrest due to hypotension/shock, pre-existing pulmonary hypertension, and prior IHCA. These covariates were selected based on a *priori* hypotheses that they would be associated with the provider’s decision to administer vasopressin within our institution and with the time to ROSC. Patients who died during the 20-min window, achieved event survival through ECPR rather than ROSC during the 20-min window, or achieved ROSC after 20 min were censored at 20 min post-dose in the Cox regression analysis. This censoring approach ensures that these patients remain at risk in the analysis, thereby providing a more accurate estimation of the treatment association with ROSC within the specified timeframe. Event outcomes of ROSC, ECPR, or death were reported separately for each treatment group.

For the secondary analysis of physiologic responsiveness, each patient was dichotomized as a responder or non-responder. The average DBP per 15-s epoch around the dose of interest was plotted for the vasopressin and matched epinephrine cohorts. The DBP response to each vasopressor was further analyzed using a regression discontinuity analysis. For the regression discontinuity analysis, the vasopressor was used as the treatment group, and DBP was the running variable. The minute of the matched vasopressor administration was used as the cutoff with a 2-min bandwidth before and a 5-min bandwidth after the matched dose. The full minute of dose administration was treated as the cutoff because the dose could have been administered during any of the four 15-s epochs during that minute. All analyses were performed with Stata version 18.0 (StataCorp, College Station, TX), and two-sided *p* values < 0.05 were considered statistically significant.

## Results

**Fig. 1:**
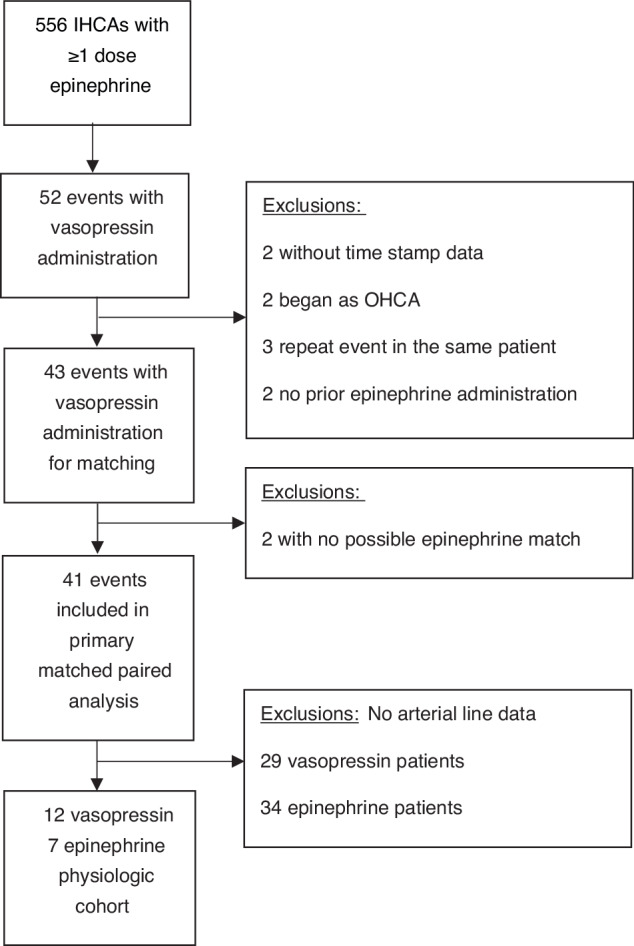
CONSORT style diagram showing serial exclusions.

Among 556 patients who had an IHCA with ≥1 dose epinephrine, 52 subsequently received vasopressin, of whom 41 met the inclusion criteria and were matched with epinephrine patients. Arterial BP waveform data were evaluable in 12/41 (29%) vasopressin and 7/41 (17%) matched epinephrine patients for inclusion in the secondary physiologic cohort data analysis (Fig. 1). There were no statistically significant differences in demographics and pre-existing conditions between the matched cohorts (Table 1), although 15/41 (37%) vasopressin patients had a prior IHCA during the hospitalization compared with 7/41 (17%) matched epinephrine patients (*p* = 0.08).

**Table 1:** Demographics and pre-existing conditions.

	Total	Vaso cohort	Epi cohort	*p* value
	*N* = 82	*N* = 41	*N* = 41	
Age (years)	1.1 (0.6–4.4)	1.0 (0.6–5.4)	1.2 (0.3–4.4)	0.65
Weight (kg)	9.4 (6.0–15.3)	9.5 (6.0–16.6)	9.0 (6.1–15.3)	0.97
Sex				0.66
Male	47 (57.3%)	22 (53.7%)	25 (61.0%)	
Female	35 (42.7%)	19 (46.3%)	16 (39.0%)	
Race				0.31
Asian	1 (1.2%)	1 (2.4%)	0 (0.0%)	
Black or African American	25 (30.5%)	11 (26.8%)	14 (34.1%)	
White	29 (35.4%)	12 (29.3%)	17 (41.5%)	
Multiple	1 (1.2%)	1 (2.4%)	0 (0.0%)	
Other	24 (29.3%)	14 (34.1%)	10 (24.4%)	
Unknown	2 (2.4%)	2 (4.9%)	0 (0.0%)	
Illness category				1.00
Cardiac	25 (30.5%)	13 (31.7%)	12 (29.3%)	
Non-cardiac	57 (69.5%)	28 (68.3%)	29 (70.7%)	
Prior IHCA^a^	22 (26.8%)	15 (36.6%)	7 (17.1%)	0.08
Prior OHCA^a^	7 (8.5%)	1 (2.4%)	6 (14.6%)	0.11
**Pre-existing conditions**				
Cancer	10 (12.2%)	6 (14.6%)	4 (9.8%)	0.74
Cardiac disease	45 (54.9%)	25 (61.0%)	20 (48.8%)	0.37
Congenital non-cardiac	16 (19.5%)	7 (17.1%)	9 (22.0%)	0.78
Genetic diagnosis	17 (20.7%)	12 (29.3%)	5 (12.2%)	0.10
Sepsis	26 (31.7%)	16 (39.0%)	10 (24.4%)	0.24
Shock/Hypotension	47 (57.3%)	27 (65.9%)	20 (48.8%)	0.18
Pulmonary hypertension	15 (18.3%)	10 (24.4%)	5 (12.2%)	0.25
Respiratory disease	69 (84.1%)	36 (87.8%)	33 (80.5%)	0.55
Neurologic disease	38 (46.3%)	22 (53.7%)	16 (39.0%)	0.27
Renal disease	20 (24.4%)	11 (26.8%)	9 (22.0%)	0.80
Hepatic disease	12 (14.6%)	5 (12.2%)	7 (17.1%)	0.76
Trauma	2 (2.4%)	0 (0%)	2 (4.9%)	0.49

Data are presented as median (IQR) for continuous measures, and *n* (%) for categorical measures.

Statistical tests: Fisher’s exact for categorical data and Wilcoxon rank-sum for continuous data.

^a^Data for prior IHCA and OHCA are for the current admission.

There were no significant differences in arrest interventions and event outcomes between groups (Table 2). Within the matched cohort, the majority had either an endotracheal tube (67/82) or a tracheostomy tube (14/82) in place at the time of arrest, and many were receiving continuous vasoactive support (54/82). The median CPR duration was 36.5 [IQR 23, 48] minutes, and the median time to study dose (either vasopressin or epinephrine) was 14.5 [10.8, 19] minutes. The median number of epinephrine doses prior to the study dose was 4 [3, 5] doses. The primary outcome of ROSC occurred in 10/41 (24%) vasopressin patients and 15/41 (36%) epinephrine patients (*p* = 0.34). An event outcome of ECPR occurred in 9/41 (22%) vasopressin patients vs. 16/41 (39%) epinephrine patients (*p* = 0.10), and vasopressin patients more frequently had an event outcome of death (22/41 [54%] vs. 10/41 [24%]; *p* = 0.01). The overall survival to discharge in the full cohort was 12% with 4/41 (10%) vasopressin patients and 6/41 (14%) epinephrine patients surviving to discharge (*p* = 0.74). After adjustment for potential confounders, the time to ROSC between the vasopressin and matched epinephrine patients was not significantly different (aHR 0.73 [95% CI: 0.31–1.7; *p* = 0.47]).

**Table 2:** Arrest characteristics and outcomes.

	Total	Vaso cohort	Epi cohort	*p* value
	*N* = 82	*N* = 41	*N* = 41	
Time to dose	14.5 (10.8–19.0)	15.0 (10.0–19.0)	14.0 (11.6–17.0)	0.80
Number of preceding epi doses	4.0 (3.0–5.0)	4.0 (3.0–5.0)	4.0 (3.0–5.0)	1.00
Cause of arrest				0.14
Respiratory decompensation	25 (30.5%)	12 (29.3%)	13 (31.7%)	
Arrhythmia/conduction defect	3 (3.7%)	0 (0.0%)	3 (7.3%)	
Hypotension/shock	52 (63.4%)	29 (70.7%)	23 (56.1%)	
Missing	2 (2.4%)	0 (0.0%)	2 (4.9%)	
Pre-arrest epi bolus	27 (32.9%)	15 (36.6%)	12 (29.3%)	0.49
Number of pre-arrest epi	2.5 (1.0–7.0)	2.5 (1.0–7.0)	2.5 (1.5–7.0)	1.00
**Pre-arrest vasoactive infusions**				
Epinephrine	33 (40.2%)	20 (48.8%)	13 (31.7%)	0.18
Dopamine	16 (19.5%)	6 (14.6%)	10 (24.4%)	0.40
Norepinephrine	14 (17.1%)	7 (17.1%)	7 (17.1%)	1.00
Vasopressin	6 (7.3%)	1 (2.4%)	5 (12.2%)	0.20
Milrinone	15 (18.3%)	7 (17.1%)	8 (19.5%)	1.00
Phenylephrine	0 (0.0%)	0 (0.0%)	0 (0.0%)	1.00
**Arrest monitoring**				
Arterial Line	37 (45.1%)	20 (48.8%)	17 (41.5%)	0.66
End tidal CO_2_	56 (68.3%)	32 (78.0%)	24 (58.5%)	0.10
CPR mechanics device	51 (62.2%)	25 (61.0%)	26 (63.4%)	1.00
**Invasive mechanical ventilation**				
Endotracheal tube	67 (81.7%)	31 (75.6%)	36 (87.8%)	0.25
Tracheostomy	14 (17.1%)	10 (24.4%)	4 (9.8%)	0.14
**Intra-arrest medications**				
Vasoactive infusion	54 (65.9%)	30 (73.2%)	24 (58.5%)	0.24
Calcium bolus	59 (72.0%)	29 (70.7%)	30 (73.2%)	1.00
Sodium bicarbonate	64 (78.0%)	34 (82.9%)	30 (73.2%)	0.42
Atropine	5 (6.1%)	4 (9.8%)	1 (2.4%)	0.36
Number of code epinephrine	7.5 (5.0–11.0)	8.0 (5.0–11.0)	7.0 (5.0–11.0)	0.67
CPR duration	36.5 (23.0–48.0)	37.0 (27.4–45.0)	36.0 (20.0–54.0)	0.73
**Event outcome**				
ROSC	25 (30.5%)	10 (24.4%)	15 (36.6%)	0.34
ECPR	25 (30.5%)	9 (22.0%)	16 (39.0%)	0.10
Death	32 (39%)	22 (54%)	10 (24%)	0.01
Survival to Hospital Discharge	10 (12.2%)	4 (9.8%)	6 (14.6%)	0.74

Data are presented as median (IQR) for continuous measures, and *n* (%) for categorical measures.

Statistical tests: Fisher’s exact for categorical data and Wilcoxon rank-sum for continuous data.

**Fig. 2:**
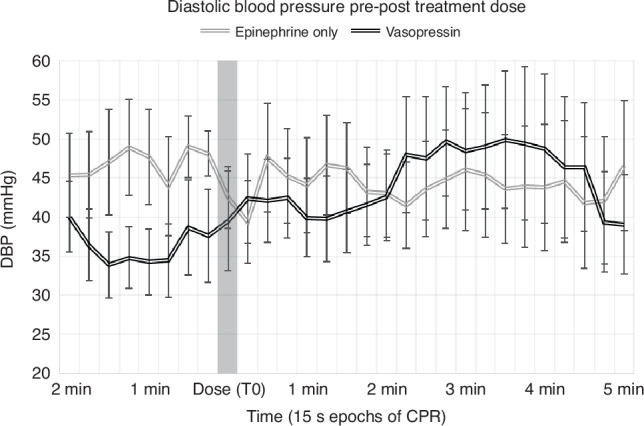
Mean DBP by treatment groups with standard error bars is plotted 2 min before and 5 min after the minute of dose administration. The minute of administration is indicated with the gray shading.

**Fig. 3:**
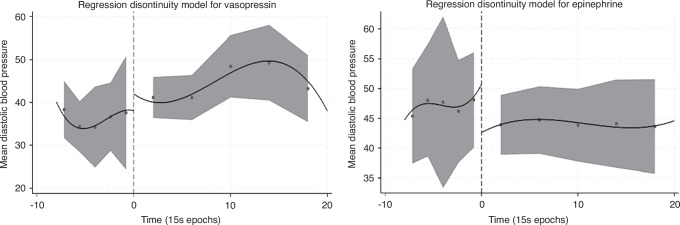
The regression discontinuity plot shows the average treatment effect on DBP around the administration of the study dose at time zero by treatment group.

For patients with intra-arrest physiologic data, the mean DBP before and after dose administration, separated by treatment group, is displayed in Fig. 2. Regression discontinuity analyses, plotted in Fig. 3, showed a change in DBP of +2.3 (95% CI: −11.4, 16.0) mmHg after vasopressin and −5.67 (−15.13, 3.80) mmHg after epinephrine. Individual patient vasopressor responsiveness, defined by an increase in mean DBP ≥ 5 mmHg, occurred in 4/12 (33%) vasopressin and 1/7 (14%) epinephrine patients (*p* = 0.60). Demographics, patient characteristics, and arrest characteristics of the vasopressin responders compared to non-responders are displayed in Table 3. All vasopressin patients with available physiologic data experienced cardiac arrest due to hypotension/shock. Two of the four vasopressin responders (50%) experienced ROSC compared to 1/8 (13%) of non-responders (*p* = 0.24). The time from the vasopressin dose to the end of CPR was significantly shorter in the patients who demonstrated response to vasopressin compared to those who did not, with a median time for responders of 11.4 (5.9–16.8) minutes compared to 25.1 (19.7–32.1) minutes in non-responders (*p* = 0.04).

**Table 3:** Vasopressin responders vs non-responders.

	Total	Responder	Non-Responder	*p* value
	*N* = 12	*N* = 4	*N* = 8	
Age (years)	0.7 (0.2–2.0)	0.6 (0.2–1.3)	0.8 (0.3–2.6)	0.50
Weight (kg)	7.8 (4.5–11.2)	6.6 (3.8–9.2)	8.0 (5.1–12.9)	0.31
Illness category				0.55
Cardiac	6 (50.0%)	1 (25.0%)	5 (62.5%)	
Non-cardiac	6 (50.0%)	3 (75.0%)	3 (37.5%)	
Prior IHCA	3 (25.0%)	2 (50.0%)	1 (12.5%)	0.24
Prior OHCA	1 (8.3%)	1 (25.0%)	0 (0.0%)	0.33
**Pre-existing conditions**				
Cancer	2 (16.7%)	0 (0.0%)	2 (25.0%)	0.52
Cardiac disease	9 (75.0%)	3 (75.0%)	6 (75.0%)	1.00
Genetic syndrome	1 (8.3%)	0 (0.0%)	1 (12.5%)	1.00
Sepsis	5 (41.7%)	3 (75.0%)	2 (25.0%)	0.22
Shock/Hypotension	10 (83.3%)	4 (100.0%)	6 (75.0%)	0.52
Pulmonary hypertension	2 (16.7%)	0 (0.0%)	2 (25.0%)	0.52
Respiratory disease	10 (83.3%)	4 (100.0%)	6 (75.0%)	0.52
Neurologic disease	3 (25.0%)	0 (0.0%)	3 (37.5%)	0.49
Renal insufficiency	7 (58.3%)	2 (50.0%)	5 (62.5%)	1.00
Hepatic insufficiency	1 (8.3%)	0 (0.0%)	1 (12.5%)	1.00
**Arrest characteristics**				
First rhythm: pulseless	8 (66.7%)	3 (75.0%)	5 (62.5%)	1.00
Time to vasopressin	10.4 (8.5–15.5)	12.0 (6.9–20.5)	10.4 (9.5–13.5)	0.86
Epinephrine bolus prior to arrest	8 (66.7%)	3 (75.0%)	5 (62.5%)	1.00
Arrest cause: hypotension/shock	12 (100.0%)	4 (100.0%)	8 (100.0%)	
**CPR medications**				
Vasoactive infusion	12 (100.0%)	4 (100.0%)	8 (100.0%)	
Calcium bolus	8 (66.7%)	1 (25.0%)	7 (87.5%)	0.07
Sodium bicarbonate	7 (58.3%)	1 (25.0%)	6 (75.0%)	0.22
Atropine	2 (16.7%)	2 (50.0%)	0 (0.0%)	0.09
Total # code epi	6.5 (4.0–9.0)	5.0 (3.5–7.5)	7.5 (5.0–9.5)	0.30
**Event outcome**				
CPR duration	32.5 (26.1–41.5)	26.1 (17.9–32.2)	36.0 (28.7–46.1)	0.13
Time to CPR end from Vaso	21.3 (11.4–27.1)	11.4 (5.9–16.8)	25.1 (19.7–32.1)	0.04
Outcome ECPR	5 (41.7%)	1 (25.0%)	4 (50.0%)	0.58
Outcome ROSC	3 (25.0%)	2 (50.0%)	1 (12.5%)	0.24
Survival to discharge	1 (8.3%)	0 (0.0%)	1 (12.5%)	1.00

Data are presented as median (IQR) for continuous measures, and *n* (%) for categorical measures.

Statistical tests: Fisher’s exact for categorical data and Wilcoxon rank-sum for continuous data.

## Discussion

In this small single-center cohort of pediatric IHCA patients who received intra-arrest epinephrine and at least one additional dose of a vasopressor medication, we did not detect a statistically significant difference in time to ROSC following vasopressin compared to matched epinephrine dosing. In a secondary analysis of the physiologic response to bolus vasopressor during IHCA, on average, neither vasopressin nor dose- and time-matched epinephrine was associated with a significant increase in DBP. However, on an individual level, 4/12 (33%) patients experienced at least a 5 mmHg rise in DBP following vasopressin administration, compared to 1/7 (14%) epinephrine patients. Collectively, these data indicate that vasopressin administered late in IHCA (median time from CPR start of 15 min with a median of four preceding epinephrine doses) did not improve clinical outcomes compared with ongoing epinephrine administration for IHCA patients, but whether vasopressin may provide physiologic improvements in a subset of patients remains unknown.

Epinephrine is the only recommended vasopressor in pediatric cardiac arrest guidelines.^5,18^ Delays in the time to administration of the first dose of epinephrine have been associated with worse survival outcomes.^19^ However, prior work has demonstrated heterogeneity in the physiologic response to the first dose of epinephrine during pediatric IHCA, with only 45% of patients achieving a DBP increase of ≥5 mmHg. Patients failing to attain this increase in DBP in response to epinephrine have inferior survival outcomes compared to those who do respond.^6^ Yet the limited published data informing cardiac arrest medication management following the first dose of epinephrine for children do not provide consistent, clear guidance.^20–23^ As vasopressin has an alternative mechanism of action for increasing aortic DBP, acting at the V1 receptor rather than α-adrenergic receptors,^13^ it is reasonable to hypothesize that it may have physiologic efficacy in some patients who fail to respond to epinephrine. Our data suggest that vasopressin may be an alternative therapy to raise DBP and facilitate ROSC in some patients who do not achieve ROSC after the first dose of epinephrine.

The published use of vasopressin during pediatric cardiac arrest is limited.^14–16,24^ Large multicenter studies report the use of vasopressin in 3–5% of all children with IHCA.^15,25^ Prior studies have not shown a significant association between the use of vasopressin and survival to discharge. The results of small prospective trials are mixed. A small pediatric feasibility trial showed higher rates of 24-h survival with the use of vasopressin; however, that study was not powered to detect differences in survival to hospital discharge or survival 30 days post-arrest.^14^ A systematic review to assess the effect of vasopressin use in adults failed to demonstrate improved outcomes.^26^ The largest retrospective review of vasopressin use during pediatric cardiac arrest identified a negative association with ROSC, but acknowledged significant limitations due to confounding by indication and the inability to determine the time of vasopressin administration relative to CPR start.^15^ Like prior observational studies, our study found that vasopressin was administered in patients with a long median duration of CPR (36.5 min) and after failing to achieve ROSC with several doses of epinephrine (median of four). In our cohort, the median time to the matched dose of vasopressor was 14.5 min compared to a median total CPR duration of 6–11 min reported in other large cohorts of pediatric IHCA.^3,25,27^ In our matched cohort, rates of ROSC and survival to discharge were also low compared to other cohorts of pediatric IHCA. Only 31% of our full cohort of vasopressin and matched epinephrine patients achieved ROSC, and 12% survived to hospital discharge compared to 40–50% survival to discharge in other recent pediatric IHCA cohorts.^28,29^

The vasopressin cohort was less likely to receive ECPR, suggesting that vasopressin may have been preferentially used in patients perceived to have limited survival potential or who were not considered ECPR candidates. This likely reflects residual confounding by indication that matching on age, illness category, and number/timing of epinephrine doses could not eliminate. These unmeasured differences limit our ability to limit our ability to infer causality regarding the effect of vasopressin on ROSC or survival. Our findings demonstrate that vasopressin use during pediatric CPR at our institution is generally reserved for patients who fail to achieve ROSC after standard care, is often given late in CPR, and is perhaps more preferentially administered when not proceeding with ECPR. Potential benefits from vasopressin may be missed within the current frame of practice.^30^

Our evaluation of the physiologic response to vasopressin administration was limited by the small sample size of patients with evaluable peri-vasopressor arterial waveform data. Within this cohort, 33% of patients experienced an increase in mean DBP ≥ 5 mmHg following vasopressin. However, these findings were more promising than those pertaining to the matched epinephrine patients, for whom mean DBP decreased on average, and among whom only one patient achieved a DBP increase of at least 5 mmHg. These findings are similar to a laboratory study in a porcine model of pediatric IHCA in which 9/19 (47%) swine that failed to achieve the goal CoPP with epinephrine reached the threshold after subsequent vasopressin.^10^ The timing of vasopressin during CPR, the pre-vasopressin DBP, and the heterogeneity of responsiveness likely contributed to the non-significant increase in DBP in our regression discontinuity analysis. Compared to this laboratory investigation, the high pre-dose DBP observed in most patients in our cohort, frequently exceeding established thresholds associated with outcomes, potentially reduced the physiologic margin for further DBP improvement, thereby precluding detection of a treatment effect. Additionally, our study assessed vasopressin administration at a single standard dose (0.4 units/kg). Whether earlier or repeated dosing, or alternative dosing strategies, might elicit more consistent physiologic responses remains unknown and warrants further investigation. Collectively, these data suggest that “rescue” vasopressin is unlikely to universally improve DBP in all epinephrine non-responders but may have a physiologic effect in a subset of such patients. Notably, all of the patients in this cohort were in an ICU at the time of arrest, most were already on a vasoactive infusion, and shock was the most common cause of cardiac arrest. We postulate that this patient population, particularly those with catecholamine-refractory shock, represents a group that could most benefit from a vasopressor with an alternative mechanism to epinephrine. However, the overall physiologic status of such patients may limit the number of patients in whom any vasopressor would be physiologically efficacious, particularly late in the course of CPR. Future study into the physiologic response to vasopressin in a larger cohort of pediatric patients, in those who fail to meet the goal DBP for age, and at earlier timepoints is necessary. Exploration into differences in the DBP response to vasopressin based on demographics, underlying conditions, or specific pre-arrest physiologic states that may identify patients for whom vasopressin could offer benefit.

The limitations of this study are important to consider in the interpretation of the results. First, this study was limited by a small sample size, which precluded our ability to provide an in-depth analysis of vasopressin responders. Using this highly granular single-center data allowed for a robust process of matching patients based on dose timing and number of doses, as well as the ability to analyze DBP data from the complete event around the time of the dose. However, the small number of patients necessarily relegates our findings to preliminary data for future investigation into the physiologic effects of vasopressin on DBP during cardiac arrest. A multicenter study will be necessary to recruit adequate subjects. Additionally, while this analysis focused specifically on DBP changes around medication administration, it did not account for other interventions or patient factors that could have contributed to the change in DBP. Second, despite matching patients as well as addressing select confounders in our primary time-to-event model, unmeasured confounding by indication continues to limit our ability to assess the association between vasopressin and ROSC. Notably, only 52/556 (9%) IHCA patients who received at least one dose of epinephrine in our database received vasopressin, whereas 34/168 (20%) patients with ≥30 min of CPR received vasopressin, suggesting potential confounding by indication. It appears that clinicians used vasopressin for patients they felt were especially unlikely to survive with standard repeated epinephrine administration and whom they deemed poor candidates for other rescue therapies such as ECPR. Prospective pediatric trials of vasopressin in epinephrine non-responders are needed to fully address this limitation. Third, the high mean pre-dose DBP in our study cohort may have diluted the physiologic effectiveness of both vasopressin and epinephrine in this cohort. The proposed mechanism of achieving ROSC through vasopressin is through an increase in DBP above specified thresholds of ≥25 mmHg in infants and ≥30 mmHg in children.^2,3^ Within this observational cohort, many patients were above these DBP thresholds prior to the study dose administration. To truly assess a clinically important response to vasopressin, we would ideally select a cohort of patients with diastolic hypotension in need of a vasopressor. Finally, inaccuracies in the recording of dose times could limit the interpretation of our results. However, all code documentation was performed by trained documenters, and relevant times, including the start of CPR and dose times, were obtained from clocks synchronized to the bedside monitor, which captured the physiologic data. The code sheet data, as well as physiologic data, were also reviewed by the investigator team at the time of entry into the database for suspected inaccuracies.

## Conclusion

In this small exploratory study of IHCA patients receiving at least one dose of a vasopressor after an initial dose of epinephrine during CPR (median 4 doses), there was no statistically significant difference in time to ROSC after vasopressin compared to epinephrine. While some patients demonstrated a physiologic DBP response to vasopressin, the residual presence of confounding by indication limits the comparison between treatments. These preliminary findings support the need for larger prospective studies to evaluate vasopressin as a potential rescue therapy in epinephrine non-responders in a larger IHCA sample size, perhaps earlier in the resuscitation period.

## Supplementary information

Supplementary Fig. S1

## Data Availability

The datasets generated during and/or analyzed during the current study are available from the corresponding author on reasonable request.
