# Supplementary material for: Comparison of vasopressin to epinephrine during pediatric in-hospital cardiac arrest: survival and physiologic responsiveness
Source: Pediatr Res. 2025 Oct 4;99(5):1872–9. doi: 10.1038/s41390-025-04374-6 (PMC12841233; doi:10.1038/s41390-025-04374-6)
Supplement: Supplementary file 1 — Supplementary Fig. S1 [file 41390_2025_4374_MOESM1_ESM.pdf]

Supplemental Figure S1. Physiologic DBP Inclusion and Exclusion Criteria

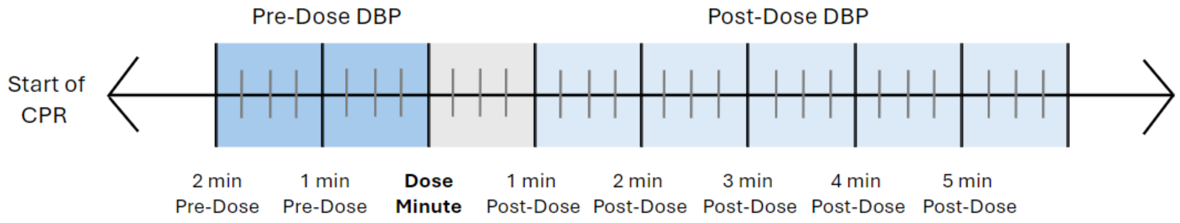

In the analysis of the physiologic cohort, evaluable DBP data from two minutes pre-dose was eligible for inclusion as the pre-dose DBP. All four epochs of data during the minute of administration were excluded from the DBP analysis as the time of administration. Evaluable DBP data in the five minutes of CPR following dose administration were eligible for inclusion until the timing of the next dose of vasopressor.
